# MiR-339-5p Regulates the Growth, Colony Formation and Metastasis of Colorectal Cancer Cells by Targeting PRL-1

**DOI:** 10.1371/journal.pone.0063142

**Published:** 2013-05-16

**Authors:** Chang Zhou, Guobing Liu, Lijing Wang, Yanxia Lu, Li Yuan, Lin Zheng, Fang Chen, Fanli Peng, Xuenong Li

**Affiliations:** 1 Department of Pathology and Key Laboratory of Molecule Tumor Pathology of Guangdong Province, Southern Medical University, Guangzhou, Guangdong, China; 2 Department of Anatomy and Histology, Guangdong Pharmaceutical University, Guangzhou, Guangdong, China; 3 Department of Obstetrics and Gynecology, Nanfang Hospital, Southern Medical University, Guangzhou, Guangdong, China; 4 Vascular Biology Research Institute, Guangdong Pharmaceutical University, Guangzhou, Guangdong, China,; University of Bari & Consorzio Mario Negri Sud, Italy

## Abstract

MicroRNAs (miRNAs) have been suggested to play a vital role in regulate tumor progression and invasion. However, the expression of miR-339-5p in colorectal cancer and its effects are not known. Here, we report that miR-339-5p is a tumor suppressor by regulating expression of PRL-1. In this study, we showed that downregulated miR-339-5p levels in colorectal cancer tissues and highly invasive CRC cell lines. Furthermore, enhancing the expression of miR-339-5p inhibited CRC cell growth, migration and invasion in vitro and suppressed tumor growth in vivo. We then screened and identified a novel miR-339-5p target, phosphatases of regenerating liver-1 1 (PRL-1), and it was further confirmed by luciferase assay. Overexpression of miR-339-5p would also reduce the expression of PRL-1 mRNA and protein. The reduced PRL-1 expression was associated with low expression of phosphorylated-extracellular signal-regulatedkinase1/2 (p-ERK1/2). Conversely, reduction of miR-339-5p by inhibitors in cells stimulated these phenotypes. In conclusion, our results demonstrate that miR-339-5p functions as a tumor suppressor and plays a role in inhibiting growth and metastasis of CRC cells through targeting PRL-1 and regulating p-ERK1/2 .These findings suggest that miR-339-5p may be useful as a new potential therapeutic target for CRC.

## Introduction

Colorectal cancer is one of the most prevalent carcinomas throughout the world. Every year, more than 1 million individuals will develop colorectal cancer, and the disease-specific mortality is nearly 33% in the developed world [1]. For many decades, the depth of tumor progression and migration has been acknowledged as major prognostic factors in CRC patients [2]. The progression of this disease undergoes many decades and involves multi-step genetic events [3]. The molecular mechanisms underlying this process still can not be documented [4]. With the development of advanced genomic technology, a newly discovered class of non-coding small RNA, termed miRNAs, have attracted enormous interest in colon cancer research [5].

MicroRNAs (miRNAs) are 20–22 nucleotide short single-stranded noncoding RNAs that regulate various cell processes at post-transcriptional levels [6]. MiRNAs have impact on critical gene controlling cellular development, differentiation, proliferation, apoptosis and metabolism [7]–[9]. Rapidly emerging evidence have demonstrated potential roles of miRNAs in the pathogenesis and progression of cancer [10]. Differential expression of miRNAs between tumour tissue and normal tissue in various cancer types has suggested miRNAs can act as oncogenes and tumor suppressors [11]–[12]. For example, first cancer-related target gene of miR-21 promotes cell migration and invasion by targeting the PTEN in human hepatocellular cancer and TPM1 in breast cancer [13], [14]. On the other hand, loss of miR-143 is observed in bladder cancer, whereas enhanced expression of miR-143 induced growth suppression in bladder cancer cells through downregulation of Erk5 expression at translational level [15]. Recently, with the development of advanced miRNA serial analysis of gene expression (miRAGE), critical miRNAs expression landscape in colorectal cancer has been well documented [16]. Overexpressed miRNAs such as miR-20, miR-21, miR-17-5p, miR-181b and miR-200c have been implicated in colonic adenomas and carcinomas [17], [18]. Lower levels of miRNAs including miR-34a, miR-126, miR-143, miR-145, and miR-133b are also confirmed in colorectal cancers [19]–[22]. Recently, a microRNA arrays to compare the microRNA profiles in the CRC tissue samples of early and non-early recurrence patients reported that down-regulation of miR-339-5p expression was associated with a poor prognosis for clinical patients with colon cancer in stage II [23]. However, until now, functional evidence of miR-339-5p in colon cancer has not been well documented and their roles in colorectal cancer progression remains unclear.

In the present study, we evaluated the role of miR-339-5p in human colon carcinoma cells. We examined the expression level of miR-339-5p in human colon cancer cells and cancer tissues, and tested its effects on cell growth, cell-cycle distribution, and colony formation and invasion capacity in vitro. We administered miR-339-5p precursor to a mouse colon cancer tumor xenograft model and further demonstrated that it could suppress colon tumor growth in vivo. Furthermore, we provide underlying mechanism that miR-339-5p can inhibit human CRC proliferation and invasiveness by targeting the PRL-1 oncogene. PRL-1 was identified as a member of the family consists of three closely related molecules (PRL-1, PRL-2, and PRL-3), which constitute a novel class of protein tyro-sine phosphatase (PTP). The PRLs are among the smallest of the PTPs, having molecular masses of 20–22 kDa and consisting primarily of a catalytic domain. Substantial evidence from cell line and murine studies suggests that these genes promote tumor formation, invasion, and metastasis [24], [25]. These results indicate that miR-339-5p can function as a tumor suppressor, regulating the maintenance and progression of cancers.

## Materials and Methods

### Clinical specimens

Samples of colorectal cancer tissue and matched normal colonic mucosa of 30 patients with colorectal cancer were collected from fresh surgical specimens, frozen in liquid nitrogen, and stored at −80°C until further analysis. All tissues had been histologically confirmed to be an adenocarcinoma of the colon. Pathologic verification and classification were performed based on the system of the International Union Against Cancer. The research protocol was approved by the Ethics Committee at Nanfang Hospital, and written consent was obtained from all patients for the use of their tissues.

### Cell culture

Human embryonic kidney 293FT cells and six human colonic carcinoma cell lines HCT116, HT29, LS174T, SW480, SW620 and LOVO with differing metastatic abilities were obtained from American Type Culture Collection (ATCC). All cell lines were cultured in RPMI 1640 (Hyclone, Logan, Utah, USA) supplemented with 10% fetal bovine serum(FBS) (Gibco-BRL, Invitrogen, Paisley, UK). Cell lines were cultured at 37°C in a humidified incubator of 5% CO2.

### Construction of pre-miR-339 over-expressing constructs and establishment of stable clone

The pLVTHM vector containing a destabilized Green Fluorescent Protein (GFP) variant (kept in our lab) was used as a miR-339-5p over-expression system. The DNA encoding pre-miR-339 was PCR-amplified from human genomic DNA using forward primers (5′ CGACGCGTCGCGCCATTGCCACGGCACCAT) and the reverse primers (5′ CCATCGATGGGGCAGAAGACCCACGCATACGAGT), digested with *MluI* and *ClaI* and ligated to pLVTHM. The constructs were confirmed by direct DNA sequencing. Lentivirus was generated by co transfection of the above construct with packaging

plasmids psPAX_2_ and pMD2.G by using Lipofectamine 2000 (Invitrogen, Carlsbad, CA) into HEK293FT cells. After 48 hours of culture, the supernatant was collected and freezed at −80°C until use. SW620 cells were transduced with vector supernatant and subsequently FACS-sorted for green fluorescent protein (GFP). Confirmation of stable transfection of the plasmids was obtained using the microRNA qRT-PCR assay.

### MicroRNA mimics, siRNA transient transfection

MicroRNA mimic, miR-339-5p inhibitor and the negative control were purchased from GenePharma (Shanghai, China). Cells were plated to 50% confluency and were transfected with 200 nM microRNA mimic or miR-339-5p inhibitor (5′- CGUGAGCUCCUGGAGGACAGGGA-3′) or inhibitor-negative control (5′- CAGUACUUUGUGUAGUACAA-3′ by Lipofectamine 2000 (Invitrogen) in Opti-MEM (Invitrogen, Carlsbad, CA), according to the manufacturer's protocol. 24 or 48 hours after transfection, cells were harvested for further experiments. MicroRNA mimic were hsa-miR-339-5p mimics, sense 5′-UCCCUGUCCUCCAGGAGCUCACG-3′ and anti-sense 5′- UGAGCUCCUGGAGGACAGGGAUU-3′ and negative control, sense 5′- UUCUCCGAACGUGUCACGUTT-3′ and anti-sense 5′-ACGUGACACGUUCGGAGAATT-3′.

### RNA extraction and SYBR green quantitative PCR analysis

Total RNA was extracted from cells using Trizol reagent (Takara, Dalian, China) according to the manufacturer's instructions. For detection of miR-339-5p expression, stem–loop reverse transcription–polymerase chain reaction (RT-PCR) was performed using an All-in-OneTM miRNA quantitative RT-PCR (qRT-PCR) Detection Kit (GeneCopoeia, Rockville, MD) according to the manufacturer's instructions. In brief, 1 mg of total RNA containing small RNA extracted from tissue samples was first polyadenylated by poly(A) polymerase and then reverse transcribed to cDNA using a mixture of oligo-dT adaptor provided in the kit. Mature miR-339-5p expression in cells and tissues was detected using the Hairpin-it TM miRNAs qPCR kit (GeneCopoeia, Rockville, MD). Expression of U6 was used as an endogenous control. The PCR reaction for amplification of miR-339-5p was conducted at 95°C for 10 min, followed by 40 cycles of 95°C for 20 sec, 60°C for 20 sec and 72°C 10 sec. For PRL-1 mRNA detection, reverse transcription was performed using the Reverse Transcriptase System (Takara, Dalian, China). PRL-1 expression was measured by SYBR green qPCR assay (Takara, Dalian, China). The primer PRL-1 sequences were: (forward) 5′GACCTGGATGGGGTAAACCT3′ and (reverse) 5′ TGTGACTTCCACAGGAGCTG3′, The primer GAPDH sequences were: (forward) 5′ACCCACTCCTCCACCTTTG3′ and (reverse) 5′ CACCACCCTGTTGCTGTAG3′. SYBR PCR was performed in an ABI PRISM 7500 Fast Real-time PCR system (Applied Biosystems). Each sample was analyzed in triplicate. Data were processed using 2^−ΔΔCT^ method.

### CCK-8 cell proliferation assay

Cell proliferation rates were measured using Cell Counting Kit-8 (CCK-8) (Dojindo Laboratories, Japan). Twenty-four hours after being transfected with a miR-339-5p inhibitor or a control miRNA, HCT116 cells were seeded at 1×10^3^ per well in 96-well plate. The cell proliferation assay was performed on days 1, 2, 3 and 4. 10 µl CCK-8 reagent was added to each well then the plate was incubated for 2 h at 37°C. Before the endpoint of incubation, the absorbance was measured at 450 nm using a Vmax microplate spectrophotometer (Molecular Devices, Sunnyvale, CA). Each sample was assayed in triplicate. While SW620 were seeded in 96-well plates at 3×10^3^cells per well, and the same experiment were performed on days 1, 2, 3, 4, 5, 6 and 7. All experiments were performed in triplicate and repeated 3 times independently. The data were plotted as means ± SD of three separate experiments.

### Cell-cycle analysis

1×10^6^ cells were harvested and fixed overnight with at 4°C in 70% ethanol. After the cells were washed twice with PBS, their DNA was stained with the Cell Cycle Detection Kit (KeyGen, Nanjin, China). The samples were quantified by flow cytometry (Becton Dickinson, NJ, USA) and results were analyzed with Modfit LT software (Verity Software House, Topsham, ME, USA) according to the manufacturer's instructions..

### Plate clone formation assay

Each well of a 6-well culture plate were seeded with 2×10^2^ cells and each group contained three wells. After incubation at 37°C for 14 days, the cells were washed twice with PBS and stained with Giemsa solution. The number of colonies containing ≥50 cells was counted under a microscope using the formula: plate clone formation efficiency = (number of colonies/number of cells inoculated)×100%.

### Cell invasion and migration assay

The cell invasion and migration ability was evaluated using transwell inserts with 8 µm pores (BD Biosciences, San Jose, CA, USA). For invasion assay, 2×10^5^ cells in serum free medium were added to each upper compartment of the chamber pre-coated with matrigel matrix (BD Biosciences, San Jose, CA, USA). 600 µl 10% FBS medium was added to the matched lower chamber. Each cell group was plated in 3 duplicate wells. After incubation for 48 hours, noninvasive cells were removed from the upper surface of the transwell membrane with a cotton swab, and cells that had migrated through the membrane and stuck to the lower membrane surface were fixed with methanol, stained with Giemsa and photographed under the microscope. For migration assay, the procedures were similar, except that 2×10^5^ cells were placed into the top chamber without matrigel matrix pre-coated. Finally, the cells in lower compartment of the chamber that had invaded to the basal side of the membrane were counted using a light microscope in 5 random visual fields (×200).

### Protein isolation and western blotting

For the protein expression analyses, standard western blottings were carried out. Cultured or transfected cells were washed twice with cold phosphate-buffered saline (PBS) and were lysed on ice in RIPA buffer with 1% PMSF (KeyGen, Nanjin, China). Protein lysates were resolved on 10% SDS polyacrylamide gel, transfered to PVDF membranes and blocked in 0.1% Tween 20 and 5% skim milk protein in Tris Buffer Saline. Proteins were probed with rabbit anti-PRL-1 monoclonal antibody (1∶800, Proteintech Corporation, USA) and rabbit anti-ERK1/2, Phospho-Erk1/2 (p-ERK1/2) monoclonal antibody (1∶1000, Bioworld Corporation, USA), rabbit anti-β-tubulin antibody (1∶2000, Epitomics Corporation, USA) overnight at 4°C. The membrane was washed and visualized with horseradish peroxidase (HRP) – conjugated secondary antibodies for 1 h. Signals were detected by enhanced chemiluminescence (KeyGen, Nanjin, China).

### Luciferase reporter assay

Prediction of miR-339-5p binding sites was performed using TargetScan software (http://www.targetscan.org). A fragment of 3′UTR of PRL-1 containing the putative miR-339-5p binding site was amplified by PCR using following primers: wt-PRL-1 (forward) 5′ CCGGCTCGAGATCTCCCACATTCATACC 3′, wt-PRL-1 (reverse) 5′ TAAGCGGCCGCTTCATTAGCAGAAACCC 3′ and inserted at the *Xol I* and *NotI* restriction sites, immediately downstream of the luciferase gene in the psiCHECK™-2 vector (Promega, Madison, WI) A psiCHECK-2 construct containing 3′UTR of PRL-1 with a mutant seed sequence of miR-339-5p was also generated using the primers: mut-PRL-1 (forward) 5′ GTCAAAGGGGCCTGAGAAAAGAATG 3′, mut-PRL-1 (reverse) 5′ TAAGTTGCACCTCAGAGTGCAAACA 3′. All constructs were verified by DNA sequencing. HEK-293FT cells and HCT116 cells were seeded in 48-well plates (2×10^3^ viable cells per well). After 24 h incubation, cells were transfected with psiCHECK™-2-PRL-1 3′UTR and psiCHECK™-2-mut-PRL-1 3′UTR in combination with control oligonucleotide (final concentration of 80 nM) or mimic (80 nM) using Lipofectamine 2000 (Invitrogen) according to the manufacturer's protocol. Forty-eight hours after transfection, Luciferase activity was measured using the Dual Luciferase Reporter Assay System (Promega). Firefly luciferase activity was normalized to renilla luciferase activity for each transfected well. All transfection experiments were conducted in triplicate and repeated 3 times independently.

### In vivo tumor growth assay

4 to 6 weeks old athymic nude mice (Southern Medical Experimental Animal Center, per) were used for tumor implantation. All the animal experiments strictly adhered to the Regulations for the Administration of Affairs Concerning Experimental Animals, the Chinese national guideline for animal experiment, issued in 1988. All procedures involving animals and their care in this study were approved and performed by the Southern Medical University Institutional Animal Care and Use Committee (Permit Number: SCXK GUANGDONG 2011-0015). The cells were harvested by trypsinization, washed twice with cold serum-free medium, and re-suspended with 200 µl serum-free medium. To evaluate cancer growth in vivo, 2×10^6^ SW620/pLVTHM-pre-miR339 and SW620/pLVTHM-NC cells were independently injected subcutaneously into the back of 2 nude mice. After tumors were detected, mouse weight and tumor size were measured every 5 days. The tumor size was measured by a caliper as length×width^2^×1/2. The mean tumor volume ± SD of each group was calculated. All mice were killed 20 days after implantation. Harvested tumors were imaged immediately after sacrifice. Then these formed tumors were removed, and tumor tissues were analysed with H&E staining.

### Statistical analysis

Results of all experiments are expressed as mean ± standard deviation SD of at least 3 independent experiments. Shapiro–Wilk test was used to verify the clinical samples' distribution. Differences were analyzed using the nonparametric test Mann–Whitney–Wilcoxon. For in vitro and in vivo studies, The Student's t-test or Analysis of variance were used to compare values of test and control samples. Data were considered to be statistically significant when P<0.05(^*^).

## Results

### MiR-339-5p is downregulated in human colon cancer tissues and cells lines

**Figure 1:**
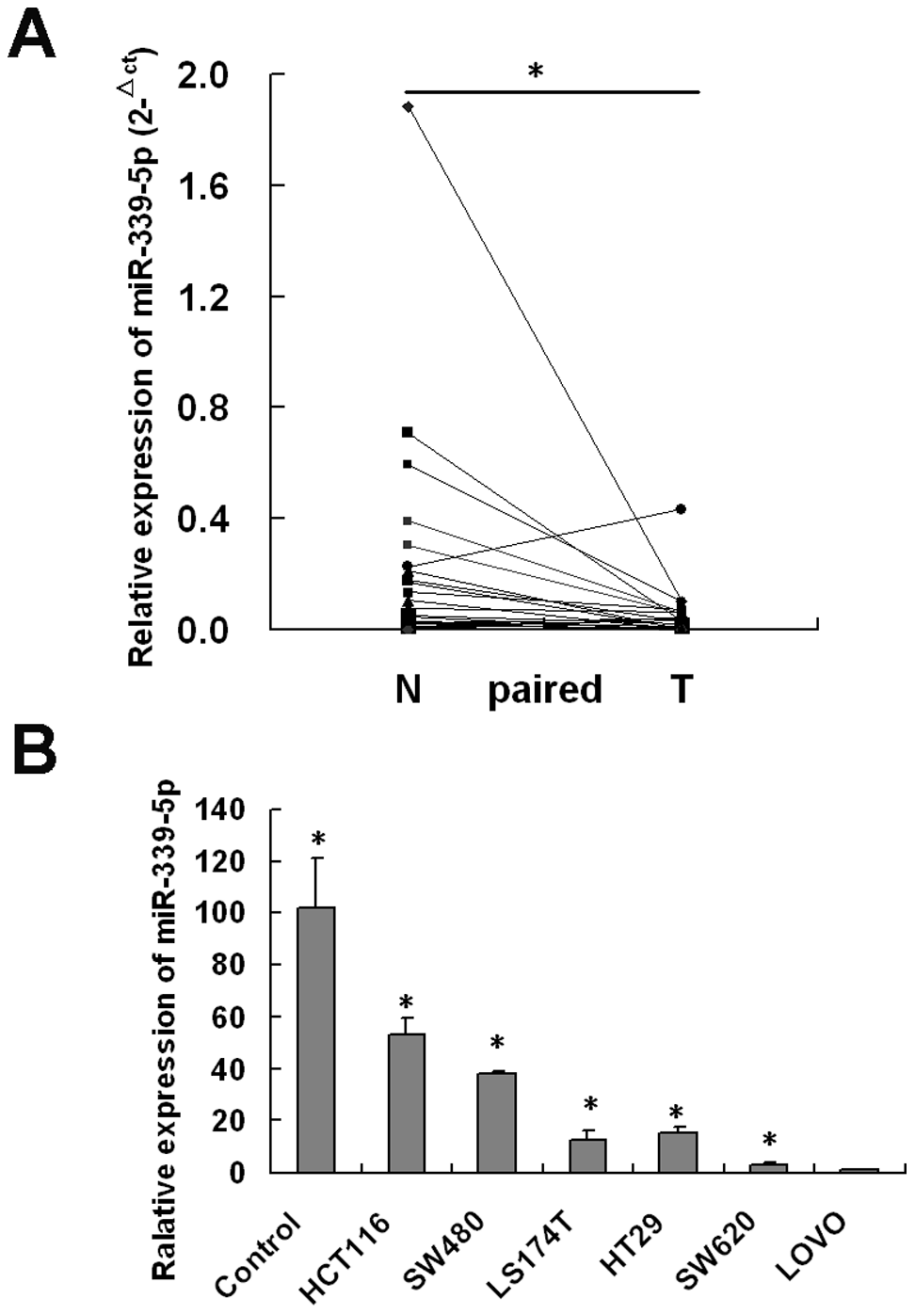
The expression levels of miR-339-5p in CRC tissues and colon cancer cell lines. (A) Expression levels of miR-339-5p were examined by qRT-PCR in 30 colon cancer tissues and their pair-matched adjacent normal colonic tissues. Each sample was analyzed in triplicate and normalized to U6 (*P<0.05). T: tumor tissues; N: adjacent normal tissues. (B) Expression levels of mature miR-339-5p were detected by qRT-PCR in six human colonic carcinoma cell lines. The relative expression of miR-339-5p was normalized to the endogenous control U6. Each sample was analyzed in triplicate. The relative miR-339-5p expression in colon cancer cell lines was much lower than that of the three non-cancerous colonic tissues (N1, N2, and N3). (*P<0.05).

To study the expression pattern of miR-339-5p, we examined miR-339-5p mRNA expression in 20 colon cancer tissues and their pair-matched adjacent normal colonic tissues using real-time quantitative RT–PCR (qRT–PCR). As shown in (Figure 1A), miR-339-5p is frequently downregulated at least twofold in CRC tissues (80%) compared with that in the paired adjacent non-tumorous tissues. In addition, we detected the expression of miR-339-5p in six human colonic carcinoma cell lines (namely HCT116, HT29, LS174T, SW480, SW620 and LOVO) by real-time quantitative reverse transcriptase-PCR (RT–PCR) and found that there are differences in the levels of miR-339-5p mRNA also existing among the six human colon cancer cell lines. Importantly, miR-339-5p was downregulated in cancer cell lines SW620 and LOVO with higher metastatic abilitity compared with HCT116, HT29, LS174T, SW480 with lower cell line (Figure 1B).

### Effect of miR-339-5p on colon cancer cells growth, migration and invasion in vitro

**Figure 2:**
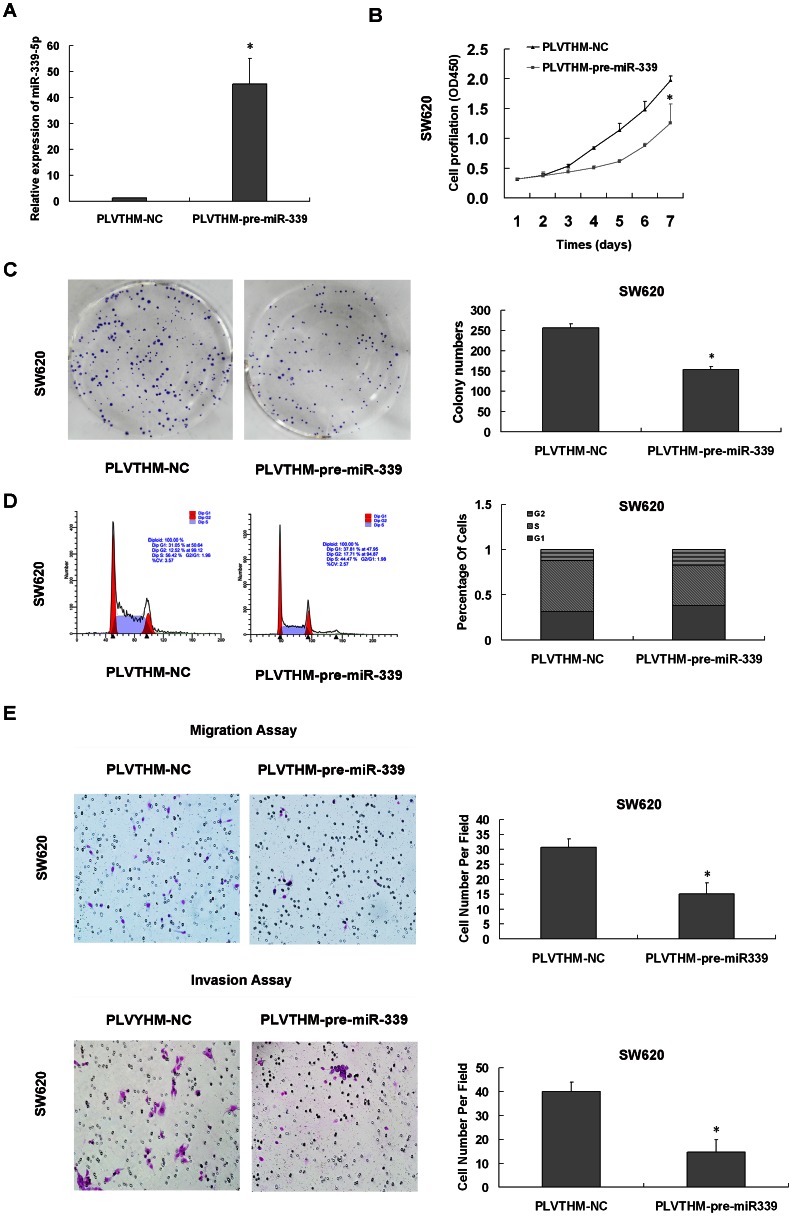
Lentivirus-mediated expression of miR-339-5p inhibited cell proliferation, migration and invasion of SW620 cells. (A) The expression of miR-339-5p was analysis in SW620 cells infected with PLVTHM-NC or pLVTHM-pre-miR-339 by qRT-PCR (*P<0.05). (B) The vitality of cells infected with PLVTHM-NC or pLVTHM-pre-miR-339 was detected using the CCK-8 assay. Values at the indicated time points were provided as the mean absorbance with an SD of five wells (*P<0.05). (C) Impact of miR-339-5p on cell cycle of SW620 cells. The percentage of cells in G1, S, and G2 phases is shown in the left panel. And the statistic analysis is also shown in the right panel. (D) Colonies formed by PLVTHM-NC or pLVTHM-pre-miR-339 infected SW620 cells were shown 2 weeks after plating. Right panel showed the quantification of the relative colony formation in PLVTHM-NC versus pLVTHM-pre-miR-339-infected cells. Values are the means ± SD of triplicate experiments (*P<0.05). (E) Transwell assay was employed to evaluate migration and invasion of SW620 cells infected with PLVTHM-NC or pLVTHM-pre-miR-339. Representative fields of migration (top) or invasive (bottom) cells on membrane (left) (Original magnification: ×200). Average number of invasive or migration cells number per field from three independent experiments ± standard (*P<0.05).

**Figure 3:**
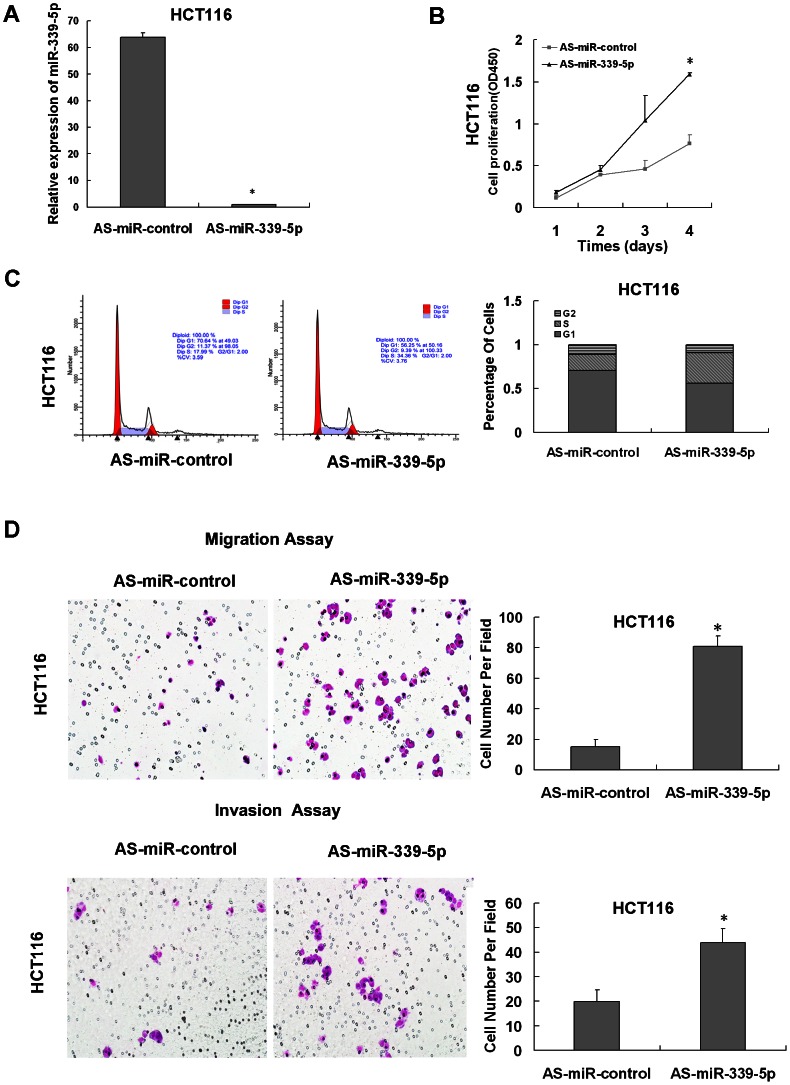
Inhibition of miR-339-5p promoted the growth, migration and invasion of HCT116 cells. (A) The expresson of miR-339-5p was analysis in HCT116 cells transfected with miR-339-5p inhibitor or negative control by qRT-PCR. (*P<0.05). (B) The vitality of cells transfected with the miR-339-5p inhibitor or negative control was detected using the CCK-8 assay. Values at the indicated time points were provided as the mean absorbance with an SD of five wells (*P<0.05). (C) Impact of miR-339-5p on cell cycle of HCT116 cells. The percentage of cells in G1, S, and G2 phases is shown in the left panel. And the statistic analysis is also shown in the right panel. (D) Transwell assay was employed to evaluate migration and invasion of HCT116 cells transfected with miR-339-5p inhibitor or negative control. Representative fields of migration (top) or invasive (bottom) cells on membrane (Original magnification: ×200). Average number of invasive or migration cells number per field from three independent experiments ± standard (*P<0.05).

To evaluate the effect of miR-339-5p on cell growth, we established a SW620 stable clone to restore the expression of pre-miR-339 in SW620 cancer cells, which had low endogenous miR-339-5p expression (Figure S1). And we tested cell proliferation rate, cell-cycle distribution and colony formation. By using qRT-PCR, we found that the expression levels of miR-339-5p were increased to about 30 folds in SW620/pLVTHM-pre-miR-339 compared with SW620/pLVTHM-NC cells (Figure 2A). As shown in Fig. 2B, the results of CCK8 assay displayed that overexpression of miR-339-5p inhibited cell proliferation in SW620 colon cancer cell. Moreover, the colony formation assay was performed to assess the long-term impact of miR-339-5p on cell growth. As shown in Figure 2C, SW620/pLVTHM-pre-miR-339 cells formed much fewer colonies than SW620/pLVTHM-NC cells. Statistical analysis indicated that increase of miR-339-5p suppressed the colony formation of SW620 cells by 34.5% (Figure 2C). We proposed that the reduced proliferation in miR-339-5p overexpressing colon cancer cells may be associated with altered cell cycle progression. The analysis of cell cycle distribution showed that miR-339-5p overexpressing SW620 cells were arreted in the G1 phase, with a corresponding reduction in the percentage of S phases (Figure 2D). To investigate if miR-339-5p regulates CRC cell migration and invasion, we performed a transwell assay to determine whether miR-339-5p was involved in the movement of colonic carcinoma cancer cells. As shown in Figure 2E, SW620/pLVTHM-pre-miR-339 cells exhibited impairment of migratory and invasive ability (reduced by 49.6% and 36.5%, respectively, P<0.05). Inversely, after transfected with miR-339-5p inhibitor, miR-339-5p expression levels were measured in HCT 116 cells (Figure 3A). Increased proliferation properties of HCT116 transfected with the miR-339-5p inhibitor were also detected by CCK-8 assay. And these transfected cells presented increased proliferative capabilities compared to negative control (Figure 3B). Furthermore, cell cycle analyses indicated reduction of cells in the G1 phase and corresponding increase cell number in S and G2 phases (Figure 3C). Correspondingly, the number of migratory and invasive HCT116 cells transfected with the miR-339-5p inhibitor was more than with the control inhibitor, showing promoted migration and invasion of the HCT116 cells by 72% and 55% (Figure 3D). These results demonstrated that miR-339-5p may control colon cancer cells proliferation and colonic carcinoma cells mobility.

### PRL-1 is a functional target for miR-339-5p in CRC cells

**Figure 4:**
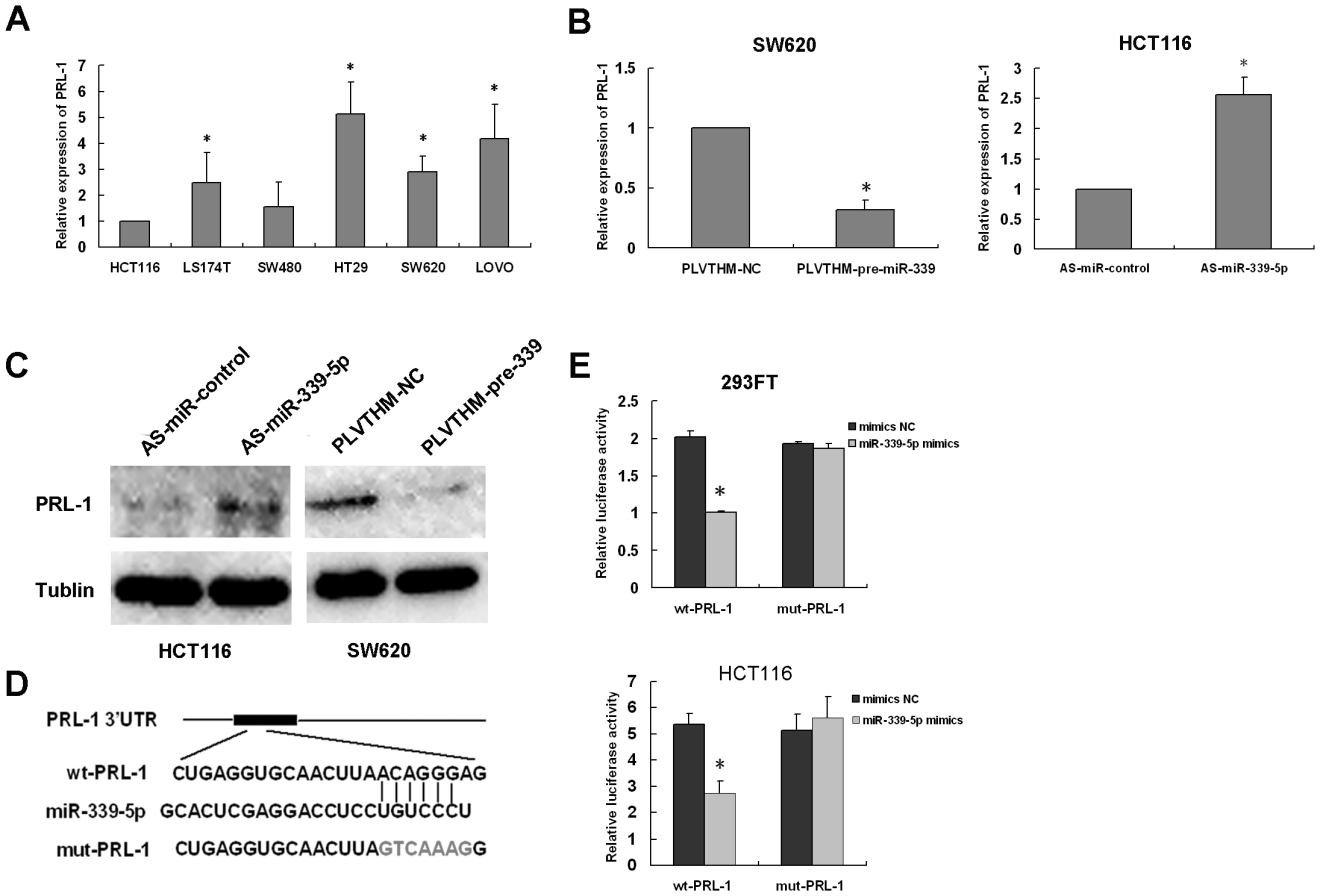
PRL-1 is a direct target of miR-339-5p in colon cancer cells. (A) Expression levels of PRL-1 were detected by qRT-PCR in six human colonic carcinoma cell lines. The relative expression of PRL-1 was normalized to the endogenous control GAPDH. Each sample was analyzed in triplicate (*P<0.05). (B) The expression of PRL-1 was analysis in SW620 cells infected with PLVTHM-NC or pLVTHM-pre-miR-339 by qRT-PCR (*P<0.05) (left). The expression of PRL-1 was analysis in HCT116 cells transfected with miR-339-5p inhibitor or negative control by qRT-PCR (*P<0.05) (right). (C) The expression levels of PRL-1 were detected using Western blot. (D) A schematic illustration of base paring between miR-339-5p and the 3′UTR of PRL-1. Substitution of seven consecutive bases (GTCAAAG to ACAGGGA) at the 3′UTR of PRL-1 for the mutant reporter construct is also shown. (E) Analysis of luciferase activity. 293 cells and HCT116 cells were co-transfected with psiCHECK™-2 luciferase reporter plasmid containing either wildtype or mutant PRL-1 3′-UTR (indicated as WT or MUT on the X-axis), and either the miR-339-5p mimics or NC mimics. Luciferase activity was assayed 48 h after transfection. Renilla luciferase activity of each sample was normalized by Firefly luciferase activity. The Y-axis represents the relative luciferase activity. Data were shown as mean ± SD from three independent experiments (*P<0.05).

**Figure 5:**
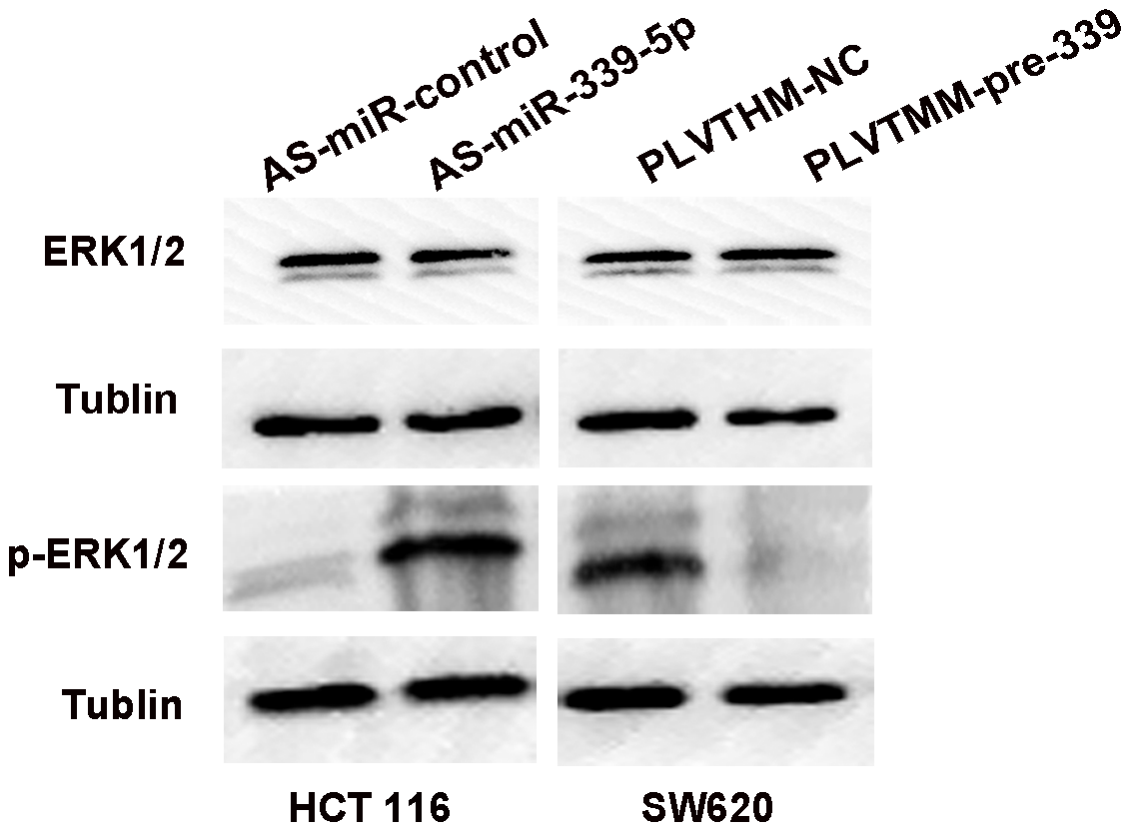
MiR-339-5p inhibited the expression of phosphorylation of Erk1/2. (A) The protein levels of Erk1/2 and phosphor-Erk1/2 were detected by Western Blot after transfection for 48 h. β-tublin was used as the loading control.

The miRNA targets predicted were obtained from miRBase Targets, TargetScan Release 5.0 and PicTar, the intersection of the three sites recommended that PRL-1 was a downstream target of miR-339-5p. To further confirm the potential relationship between miR-339-5p and the downstream gene PRL-1, we further detected the PRL-1 expression level in primary CRC tumour tissues in the same samples that mentioned in the previous experiment by qRT-PCR (Figure S2). Our results demonstrated that the slightly decrease in PRL-1 expression in tissues samples from nontumorous colon. The expression of PRL-1 was further determined in colonic carcinoma cell lines HCT116, HT29, LS174T, SW480, SW620 and LOVO (Figure 1B). Interestingly, we found opposite correlation between miR-339-5p and PRL-1 (mRNA levels) in colonic carcinoma cells. To futher confirm the potential suppressive effect of miR-339-5p on PRL-1 expression, we transfected pre-miR-339 to SW620 cells. The result showed that miR-339-5p suppressed PRL-1 mRNA and protein expression in SW620/pLVTHM-pre-miR-339 cells compared with SW620/pLVTHM-NC cells (P<0.05) (Figure 4B, Fig. 4C). Consisting with the result, both mRNA and protein levels of PRL-1 were up-regulated were observed in HCT116 cells transfected with the miR-339-5p inhibitor compared to the control inhibitor (P<0.05) (Figure 4B, Figure 4C). To clarify whether miR-339-5p directly interact with 3′-UTR region of the target genes (PRL-1) we performed luciferase reporter assays. We found that the miR-339-5p targets sequence at nt739–745 of the PRL-1 3′-UTR. A reporter plasmid driven by the sv promoter, including the 519 nt-wild-type-3′-UTR of PRL-1 was cloned. Then we cloned another reporter construct in which the conserved targeting region of miR-339-5p within nt739–745 was specifically mutated. We cotransfect miR-339-5p mimics and luciferase reporter constructs containing wild type (Figure 4D) or mutant (Figure 4D) PRL-1 3′-UTR. Luciferase activity was dramatically decreased by approximately 50% in the presence of miR-339-5p when compared with its negative miRNA control (Figure 4E). In contrast, miR-339-5p did not alter activity of the mutant PRL-1 luciferase reporter (Figure 4E), indicating miR-339-5p specifically act on wild-type PRL-1 3′-UTR. We also found that inhibition of miR-339-5p upregulated the PRL-1 expression and improved the p-Erk1/2 levels. While restoration of the expression of miR-339-5p may inhibit expression of PRL-1 and p-Erk1/2 (Figure 5).

### MiR-339-5p inhibits tumour growth in vivo

**Figure 6:**
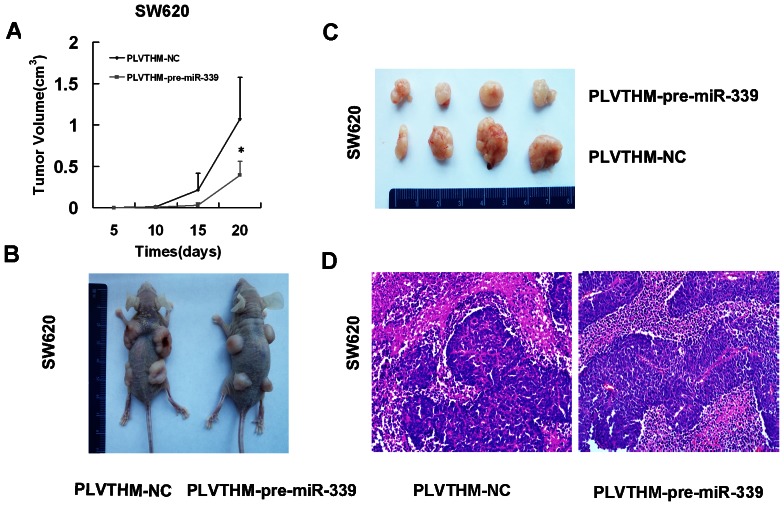
miR-339-5p inhibited colon tumor growth in vivo. (A) In all, 2×10^6^ SW620/pLVTHM-NC and SW620/pLVTHM-pre-miR339 cells were independently injected into the back of 2 nude mice. Tumor sizes were measured at different time points until day 20 when mice were killed. Mean size of the tumors per animal was plotted (*P<0.05). (B) External whole-body images of SW620/pLVTHM -NC and SW620/pLVTHM-pre-miR-339 cells mice and cancers were obtained 20 days after injection. (C) Subcutaneous tumor regeneration from SW620/pLVTHM-NC and SW620/pLVTHM-pre-miR-339 cells. (D) Representative photographs of H&E staining of primary cancer tissues are shown (magnification, ×200).

To further explore the role of miR-339-5p in tumor growth in vivo, SW620/pLVTHM-pre-miR-339 cells or SW620/pLVTHM cells were injected subcutaneously to the blank of nude mice, respectively, into nude mice. The tumour growth rate of SW620/pLVTHM-pre-miR-339 cells was slower than that of SW620/pLVTHM cells (Figure 6A). 3 weeks after injection. SW620/pLVTHM-pre-miR-339 cells formed smaller tumors than SW620/pLVTHM-NC cells in vivo and their average volume was ∼36.9% of the control group at day 20 (P<0.05) (Figure 6B, Figure 6C). Representative photographs of H&E staining of primary cancer tissues are proceeded. These results indicate miR-339-5p has a potential as a novel tumor suppressor that can suppress tumor growth (Figure 6D).

## Discussion

Accumulating studies have revealed microRNAs could influence tumor growth, invasion, angiogenesis and metastasis in hematological malignances and solid tumors including colorectal cancer. In a recent study, it has been reported that miR-339-5p may be abnormally down-regulated in colon-cancer tissue using new microRNA screening tools. However, few studies are available on its functions in colorectal cancer. Our current data showed that of miR-339-5p expression was decreased in colorectal cancer tissues and miR-339-5p was downregulated in cancer cell lines with higher metastatic abilitity compared with cancer cell lines with metastatic abilitity. The results indicated loss of miR-339-5p might be important for CSC formation and progress. And HCT116 beared wild type APC and while HT29 had mutant APC [26]. The levels of expression of mir-339-5p in HT29 are lower than in HCT116. It would be interesting to establish a correlation between APC mutations and levels of miR-339-5p. And we planed to proceed relative reseach in APC min mice.

Functional studies demonstrated overexpression of miR-339-5p inhibited colon cancer cell proliferation and colony formation, induced cell cycle arrest in G1 phase cell migration and invasion in vitro, and prevented tumor growth in vivo. Correspondingly, miR-339-5p inhibitors transfection promoted proliferation, migration and invasion of CRC cells. The results revealed miR-339-5p play a potential suppressive role in colorectal cancer.

To understand the functional mechanism of microRNAs, identifying targets involved in their regulation is important. A single miRNA might regulate many different targets. Several biological targets of miR-339-5p have been identified in previous studies. Zheng-sheng Wu et al. indicated that miR-339-5p targets BCL-6 and dramatically inhibited breast cancer cell migration and invasion in vitro [27]. In addition, it has been reported that Dicer-regulated microRNAs 222 and 339 promote resistance of cancer cells to cytotoxic T-lymphocytes by down-regulation of ICAM-1 [28]. In our study, by integrating predictions of several major network station, we identified PRL-1 as a functional downstream target of miR-339-5p. Three lines of evidence support our finding. First, stable overexpressing pre-miR-339 in SW620 cells or transient knockdown of miR-339-5p in HCT116 cells reduced or improved PRL-1 levels, respectively, at both the mRNA and protein levels. Second, mRNA expression of miR-339-5p and PRL-1were found to be inversely related in colon cancer cells. Third, overexpression of miR-339-5p decreased the luciferase activity upstream of the wild type 3′UTR of PRL-1.In contrast, However, the mutant reporter plasmid abolished miR-339-5p mimics-mediated increase in luciferase activity in both 293FT cells and HCT116 cells. Therefore, we found that miR-339-5p function depended on PRL-1 directly binding to its 3′UTR.

Emerging evidence has demonstrated the possible functions of the PRL-1 proteins in cancers [29], [30]. PRL-1 belongs to the PRL subfamily of protein tyrosine phosphatases. The subfamily also includes PRL-2 and PRL-3 [31]–[33]. PRL-1 plays a critical role in development or progression of cancers, such as cell growth, migration and invasion [34], [35], however, we also found PRL-1 downregulated in colon tissues samples compared with normal colon tissue, In fact, the expression of PRLs, reported by Ying Wang et al., was undetectable in the normal colon and in colonic adenoma tissues. PRLs were expressed strongly in all three lymph node metastases that were studied in each case. They also reported that PRLs were mainly expressed in the cytoplasm and at the cytoplasmic membranes of the colonic adenocarcinoma cells [36]. And our qRT-PCR results showed higher expression level of PRL-1 in SW620 and LOVO with high metastatic abilities than that in HCT116, LS14T, SW480 colon cancer cells with low metastatic abilities. This may support a tumor stimulation role for PRL-1 in colorectal cancer. PRL-1 protein was identified to be a specific promoter of the ERK1/2 dependent signaling pathway [37], [38]. We found that inhibition of miR-339-5p upregulated the PRL-1 expression and improved the p-Erk1/2 levels. While restoration of the expression of miR-339-5p may inhibit expression of PRL-1 and p-Erk1/2. The results indicate that miR-339-5p may participate in the modulation of malignant biological behavior. PRL-1 upregulation by miR-339-5p provides a proliferative advantage or invasion action for tumor cells by permitting activation of the ERK1/2 pathway.

In our research, we mainly discuss the functions of miR-339-5p. However, in humans, 2 different mature microRNA sequences are excised from opposite arms of the stem loop pre-miR-339 and generate 2 different microRNAs—hsa-miR-339-5p and hsa-miR-339-3p. Several reports have indicated that these 5p and 3p microRNAs, although generated from a single primary transcript, have different sequences and functions. Uchino K et al. showed that microRNA (miR)-582-5p and -3p, which were strongly decreased in high-grade bladder cancer clinical samples, both of them suppressed the expression of the same set of target genes such as protein geranylgeranyltransferase type I beta subunit (PGGT1B), leucine-rich repeat kinase 2 (LRRK2) and DIX domain containing 1 (DIXDC1) [39]. Additionally miR-34c-3p, but no miR-34c-5p inhibited cell migration and invasion in SiHa cells, which was reported. Recently, Maria I et al. reported that miR-28 suppressed proliferation but activated metastasis, because these 5p and 3p miRNAs target different mRNAs [40]. MiR-28-5p altered expression of CCND1 and HOXB3, whereas miR-28-3p bound NM23-H1 [41]. Because the differential roles of the two possible mature miRNAs are existed, we are interested in role of miR-339-3p. Now we detect the functions of miR-339-3p in vitro (data do not show), the next plan is to study the mechanism of miR-339-3p in the future.

In summary, we present evidence that miR-339-5p functions as a tumor suppressor in colorectal cancer cell lines. The effects of miR-339-5p on colon cancer cell proliferation, colony formation and migration observed in this study may be partially due to its regulation of PRL-1 and activation of ERK1/2 signalling pathway. These findings may provide the molecular mechanism of miR-339-5p in tumorigenesis and metastasis of colon adenocarcinoma. Therefore, miR-339-5p can be regarded as a new therapeutic target for colon cancer by re-expressing miR-339-5p.

## Supporting Information

Figure S1 **Light and fluorescent images of SW620/pLVTHM-pre-miR-339 after lentivirus packaging and transfection.** (Original magnification: ×200).
(TIF)

Figure S2 **The expression levels of PRL-1 in CRC tissues and colon cancer cell lines.** Expression levels of PRL-1 were examined by qRT-PCR in 30 colon cancer tissues and their pair-matched adjacent normal colonic tissues. Each sample was analyzed in triplicate and normalized to U6. T: tumor tissues; N: adjacent normal tissues.
(TIF)
