# Supplementary figures and images for: MiR-339-5p Regulates the Growth, Colony Formation and Metastasis of Colorectal Cancer Cells by Targeting PRL-1
Source: PLoS One. 2013 May 16;8(5):e63142. doi: 10.1371/journal.pone.0063142 (PMC3656035; doi:10.1371/journal.pone.0063142)

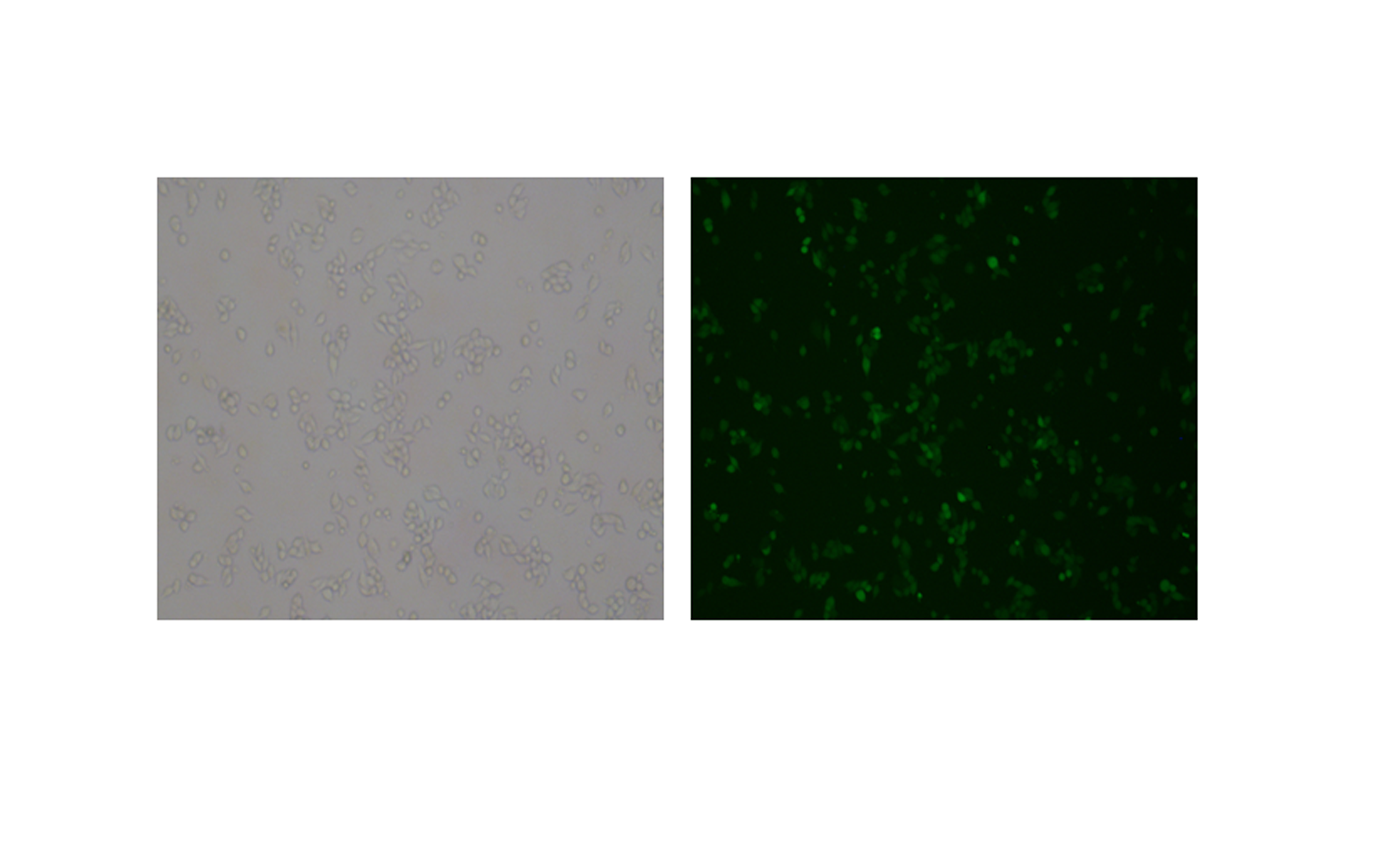

Supplement: Figure S1 — Light and fluorescent images of SW620/pLVTHM-pre-miR-339 after lentivirus packaging and transfection. (Original magnification: ×200). (TIF) [file pone.0063142.s001.tif]

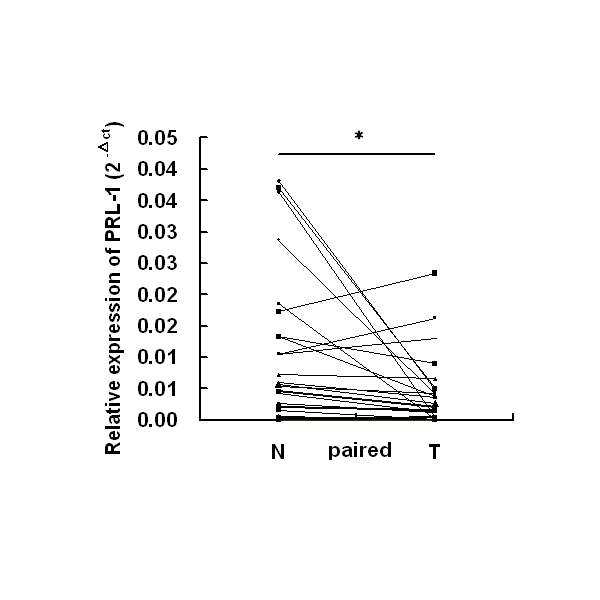

Supplement: Figure S2 — The expression levels of PRL-1 in CRC tissues and colon cancer cell lines. Expression levels of PRL-1 were examined by qRT-PCR in 30 colon cancer tissues and their pair-matched adjacent normal colonic tissues. Each sample was analyzed in triplicate and normalized to U6. T: tumor tissues; N: adjacent normal tissues. (TIF) [file pone.0063142.s002.tif]
